# EP3 Blockade Adds to the Effect of TP Deficiency in Alleviating Endothelial Dysfunction in Atherosclerotic Mouse Aortas

**DOI:** 10.3389/fphys.2019.01247

**Published:** 2019-09-26

**Authors:** Chuangjia Hu, Bin Liu, Yineng Xu, Xiangzhong Wu, Tingting Guo, Yingzhan Zhang, Jing Leng, Jiahui Ge, Gang Yu, Jinwei Guo, Yingbi Zhou

**Affiliations:** ^1^Department of Cardiology, First Affiliated Hospital, Shantou University Medical College, Shantou, China; ^2^Cardiovascular Research Center, Shantou University Medical College, Shantou, China

**Keywords:** PGI_2_, prostanoid receptors, endothelium, relaxation, contraction, atherosclerosis

## Abstract

Endothelial dysfunction, which leads to ischemic events under atherosclerotic conditions, can be attenuated by antagonizing the thromboxane-prostanoid receptor (TP) that mediates the vasoconstrictor effect of prostanoids including prostacyclin (PGI_2_). This study aimed to determine whether antagonizing the E prostanoid receptor-3 (EP3; which can also be activated by PGI_2_) adds to the above effect of TP deficiency (TP^–/–^) under atherosclerotic conditions and if so, the underlying mechanism(s). Atherosclerosis was induced in ApoE^–/–^ mice and those with ApoE^–/–^ and TP^–/–^. Here, we show that in phenylephrine pre-contracted abdominal aortic rings with atherosclerotic lesions of ApoE^–/–^/TP^–/–^ mice, although an increase of force (which was larger than that of non-atherosclerotic controls) evoked by the endothelial muscarinic agonist acetylcholine to blunt the concurrently activated relaxation in ApoE^–/–^ counterparts was largely removed, the relaxation evoked by the agonist was still smaller than that of non-atherosclerotic TP^–/–^ mice. EP3 antagonism not only increased the above relaxation, but also reversed the contractile response evoked by acetylcholine in NO synthase-inhibited atherosclerotic ApoE^–/–^/TP^–/–^ rings into a relaxation sensitive to I prostanoid receptor antagonism. In ApoE^–/–^ atherosclerotic vessels the expression of endothelial NO synthase was decreased, yet the production of PGI_2_ (which evokes contraction via both TP and EP3) evoked by acetylcholine was unaltered compared to non-atherosclerotic conditions. These results demonstrate that EP3 blockade adds to the effect of TP^–/–^ in uncovering the dilator action of natively produced PGI_2_ to alleviate endothelial dysfunction in atherosclerotic conditions.

## Introduction

The endothelium releases vasoactive substances to regulate cardiovascular function (Furchgott and Vanhoutte, 1989). Among them, NO and endothelium-derived hyperpolarizing factor (EDHF) are recognized to mediate vasodilator activities (Furchgott and Zawadzki, 1980; Komori and Vanhoutte, 1990; Feletou and Vanhoutte, 2009). In contrast, prostacyclin, also termed prostaglandin I_2_ (PGI_2_), which is derived from endothelial cyclooxygenase (COX)-catalyzed metabolism and can be released along with NO and EDHF, mediates vasodilator as well as vasoconstrictor reactions (Bunting et al., 1976; Dusting et al., 1977; Levy, 1978; Williams et al., 1994; Gluais et al., 2005; Tang et al., 2005; Liu et al., 2012a, c; Ahmetaj-Shala et al., 2015; Li et al., 2016). Notably, the initial concentration of PGI_2_ to evoke contraction is similar to that of the prostanoid (from ∼0.003 μM in pre-contracted mouse vessels) to cause relaxation (Liu et al., 2012a, 2013). It has now become clear that vasomotor reactions to PGI_2_ can be concurrently modulated by the I prostanoid receptor (IP; the prototype PGI_2_ receptor), the thromboxane (Tx) prostanoid receptor (TP; the original receptor of TxA_2_) and/or the E prostanoid receptor-3 (EP3; an original receptor of PGE_2_); hence a contraction to PGI_2_ can result from vasoconstrictor activities of TP and/or EP3 outweighing the dilator effect of concurrently activated IP (Liu et al., 2012a, c, 2017; Luo et al., 2016; Li et al., 2017). In many vascular beds, including some critical vessels, e.g., renal arteries, PGI_2_ has been recognized as an endothelium-derived contracting factor (EDCF) in health as well as in disease conditions (Liu et al., 2012b, 2013; Liu D. et al., 2015).

In vascular diseases, such as atherosclerosis and hypertension, the bioavailability of endothelial NO has been suggested to decrease (Uren et al., 1994; Holm et al., 1997; Vanhoutte, 2009, 2011). Meanwhile, the synthesis of PGI_2_ is less vulnerable to impairment caused by vascular pathologies, and sometimes it may increase in disease conditions, including atherosclerosis (FitzGerald et al., 1984; Gluais et al., 2005; Vanhoutte, 2011; Li et al., 2016). Endothelial dysfunction, which leads to serious ischemic events, can result from decreased endothelial NO bioavailability and/or increased PGI_2_’s EDCF-like action (Uren et al., 1994; Holm et al., 1997; Gluais et al., 2005; Vanhoutte, 2009, 2011; Liu D. et al., 2015; Liu et al., 2017). Accordingly, organic nitrates have been commonly used as NO donors for the pathological condition. However, the effect of such compounds could be limited by the development of tolerance (Divakaran and Loscalzo, 2017). As a result, targeting the EDCF-like action of PGI_2_ and/or uncovering its dilator function could be an alternative solution for the above-mentioned dilemma. Indeed, antagonizing TP has been found to alleviate endothelial dysfunction in atherosclerosis (Li et al., 2016; Romero et al., 2016; Huang et al., 2018). Interestingly, PGI_2_ also activates EP3, whose absence together with that of TP has been suggested to be essential for the full dilator action of PGI_2_ (Li et al., 2017; Liu et al., 2017). On the other hand, the precise involvement of EP3 in endothelium-mediated vasoresponses under atherosclerotic conditions has not been clearly elucidated. In addition, it remains to be determined whether the dilator effect of natively produced PGI_2_ adds to the endothelium-mediated dilator function of the disease condition, since EDHF, which mediates dilator activity via a pathway shared by PGI_2_, may increase (Ozkor and Quyyumi, 2011; Csanyi et al., 2012; Hellsten et al., 2012), while IP can become dysfunctional in diseases, including atherosclerosis (Gluais et al., 2005; Feletou et al., 2009; Romero et al., 2016; Liu et al., 2017).

To address the above issues, in this study atherosclerosis was induced in ApoE^–/–^ mice and those with both ApoE^–/–^ and TP^–/–^. Experiments were then performed to determine whether EP3 blockade adds to the effect of TP^–/–^ on endothelial dysfunction in atherosclerosis, and if so the underlying mechanism(s).

## Materials and Methods

### Chemicals and Solutions

The NO synthase (NOS) inhibitor N^ω^-nitro-L-arginine methyl ester (L-NAME), the endothelial muscarinic agonist acetylcholine, and the non-selective COX-inhibitor indomethacin, the EP3 receptor antagonist L798106, and the α_1_-adrenergic agonist phenylephrine were purchased from Millipore Sigma (St Louis, MO, United States). PGI_2_, the IP antagonist CAY10441, and the TP receptor antagonist SQ29548 were bought from Cayman Chemical (Ann Arbor, MI). L-NAME, phenylephrine, and acetylcholine were dissolved in distilled water, while PGI_2_ was dissolved in carbonate buffer (50 mM; pH 10.0). Indomethacin, L79816, and SQ29548 were dissolved in DMSO at 2,000-fold working concentration (the final concentration of DMSO was 0.05/100, v/v). Concentrations of indomethacin, L79816, CAY10441 and SQ29548 were used according to our previous reports, in which these inhibitors were able to abolish the effects of intended targets (Liu et al., 2012a, 2017; Li et al., 2016, 2017).

The compositions of physiological salt solution (PSS; pH 7.4 with 95%O_2_-5% CO_2_) were as follows (in mM): NaCl 123, KCl 4.7, NaHCO_3_ 15.5, KH_2_PO_4_ 1.2, MgCl_2_ 1.2, CaCl_2_ 1.25, and D-glucose 11.5. The 60 mM K^+^-PSS (K^+^) was prepared by replacing an equal molar of NaCl with KCl.

### Mice, Induction of Atherosclerosis, and Tissue Preparation

All procedures performed on mice were in conformance with the Guide for the Care and Use of Laboratory Animals published by the US National Institutes of Health (NIH Publication No. 85-23, revised 1996), and approved by the Institutional Animal Research and Use Committee of Shantou University.

C57BL/6 WT mice were bought from Vital-River (Beijing, China). ApoE^–/–^ mice were purchased from Slac (Shanghai, China), while TP^–/–^ mice were produced as described previously (Li et al., 2017). ApoE^–/–^ and TP^–/–^ mice (both were of C57BL/6 genetic background) were first cross-bred to yield apoE^±^/TP^±^ and then to apoE^–/–^/TP^–/–^ mice. Genotyping was performed by PCR of tail biopsy described previously (Li et al., 2016, 2017).

Mice were housed in the Animal Center of Shantou University Medical College (SPF grade; Temp: 20–26; Humility: 40–70%) with standard day/night (12/12) cycles and free accesses to food and drink. Atherosclerosis was induced in male apoE^–/–^ or apoE^–/–^/TP^–/–^ mice by 12 week of atherogenic diet containing 1.25% cholesterol (Research Diets Inc., New Brunswick, NJ, Canada), starting from the beginning of 9th week of age. Male WT or TP^–/–^ mice of comparable age fed on normal chow were used as non-atherosclerotic negative controls to indicate the abnormality developed in atherosclerotic conditions. Mice were killed by CO_2_ inhalation. With the help of a binocular microscope, aortas were isolated and dissected free of adherent tissues. For functional studies, the abdominal aorta was examined for spots of atherosclerotic lesions (the outer layer shows loss of gloss with grayish appearance under the light microscope) and vessels were cut into 1-mm long rings. Rings with atherosclerotic lesions were used to study vasomotor reactions of atherosclerotic conditions.

### Evaluation of Atherosclerotic Lesions

After cleaned of fat and adherent tissues, the entire aorta was opened longitudinally, and pinned out flat on a silicon plate. To visualize atherosclerotic lesions, vessels were stained with Sudan IV (Sigma, St Louis, MO, United States) and analyzed with a digitalized image system (Digital Sight, DS-U3; Nikon, Japan). Lesion area was evaluated by calculating the ratio of red-stained area to that of the whole aortas.

### Analyses of Blood Lipids

Atherosclerotic mice (12 week after atherosclerotic induction) or age-matched non-atherosclerotic controls were anesthetized with sodium pentobarbital (i.p.; 50 mg kg^–1^). Blood was collected from the right ventricle following thoracotomy, and after coagulation, serum was separated by centrifugation. Triglyceride, total cholesterol, and LDL cholesterol were analyzed using a homogenous enzymatic colorimetric assay with a C702/Cobas 8000 analyzer (Roche Diagnostic, Mannheim, Germany).

### Measurement of Systemic Blood Pressure

In some experiments, mouse blood pressure at the end of 12 week after atherosclerotic induction or that of age matched non-atherosclerotic controls was measured with a non-invasive, computerized tail-cuff system (ALC-NIBP, ALCBIO; Shanghai, China) as described previously (Zhu et al., 2014). Briefly, mice were first accustomed to tail-cuff blood pressure measurements for three consecutive days, and blood pressure was then measured on the 4th day. All measurements were performed during day time (10:00 am–4:00 pm), and in an examiner blinded manner (Zhu et al., 2014). Mean arterial blood pressure (MAP), which was taken from the averaged value of three measurements with similar values, was used for analysis.

### Analyses of Vasomotor Reactions

Analyses of vascular function were performed as described elsewhere (Liu et al., 2012a; Li et al., 2016). Briefly, the vascular ring was mounted between two tungsten wires in an organ bath filled with PSS aerated with 95% O_2_-5% CO_2_ and maintained at 37°C. One wire was stationary, whereas the other was connected to an AE801 force transducer (Kronex, Oakland, CA, United States). Thereafter, vessels were stimulated with 60 mM K^+^ every 15 min, and the resting tension was adjusted stepwise to an optimal level (∼300 mg), at which point the response to 60 mM K^+^ was maximal and reproducible.

In some experiments, vessels were removed of the influence of endothelial NO by the treatment with L-NAME (1 mM), under which the response of arteries appears similar to that of eNOS^–/–^ mice (Zhou et al., 2005). All inhibitors were added 30 min before the vessel was contracted with an agonist, and kept in the solution throughout the experiment. For control responses, the inhibitor was replaced with vehicle alone. The response to an agonist under the baseline conditions (relaxed in PSS) was expressed relative to the contraction evoked by 60 mM K^+^, while that during the contraction evoked by phenylephrine (30 or 2 μM in intact or NOS-inhibited conditions, respectively, to evoke a sustained contraction ∼80–100% that of 60 mM K^+^) was expressed as a change of force (Δforce) relative to the value of phenylephrine -induced force immediately before the application of the agent.

### Western Blots

Proteins of eNOS, PGI_2_ synthase (PGIS), IP, and β-actin (internal controls) in abdominal aortas were detected by Western blots. Briefly, vessels were minced in ice-cold RIPA buffer containing proteinase inhibitor cocktail (Roche Applied Science, Mannheim, Germany), followed by homogenizing using a glass homogenizer. Total proteins were separated by 10% SDS-PAGE and transferred to a nitrocellulose membrane, which was then sequentially probed with respective first and secondary antibodies. Anti-eNOS antibody (rabbit polyclonal; 1:1,000 dilution) was purchased from Abcam (Cambridge, MA, United States), while anti-PGIS (rabbit polyclonal; 1:2,000 dilution) and anti-IP (rabbit polyclonal; 1:2,000 dilution) antibodies were bought from Cayman Chemical (Ann Arbor, MI, United States). Anti-β-actin (rabbit polyclonal; 1:5,000 dilution) antibody was bought from Santa Cruz Biotechnology (Santa Cruz, CA, United States). Immunocomplexes were visualized with SuperSignal West Femto maximum sensitivity substrate (Pierce, Rockford, IL, United States) and detected by ChemiDoc XRS+ chemiluminescence imager (Bio-Rad, Hercules, CA, United States). Band densities, which were analyzed by Image Lab software version 4.1 (Bio-Rad), were expressed relative to β-actin and normalized by the average value of controls.

### Assay of the PGI_2_ Metabolite 6-keto-PGF_1α_

The PGI_2_ metabolite 6-keto-PGF_1α_ produced in abdominal aortas was measured with an EIA kit (Cayman Chemical). Briefly, after being cut open and rinsed of blood components, abdominal aortas were incubated with PSS at 37^*o*^C for 30 min, followed by exposures to PSS (200 μl; 37^*o*^C) and that containing acetylcholine (10 μM) sequentially for 15 min each. Thereafter, vessels were transferred and weighed, while the reaction solutions (2 μl) were taken for 6-keto-PGF_1α_ measurement, according to instructions of the manufacturer. The amount of 6-keto-PGF_1α_ was expressed in ng per mg of wet tissue.

### Real-Time PCR

Expressions of TP, EP3 and β-actin (internal control) in abdominal aortas were analyzed by real-time PCR. Total RNA was prepared with RNAiso Plus solution (TaKaRa, Dalian, China) according to the manufacturer’s instruction. First-strand cDNA was synthesized using total RNA (250 ng) and oligo(dT)15 primers (TaKaRa; Dalian, China). Primers for TP, EP3 and β-actin were as described elsewhere previously (Liu et al., 2013; Li et al., 2017). Real-time PCR was performed using a SYBR PrimScript RT-PCR kit (Thermo Fisher Scientific, Carlsbad, CA, United States). The amount of mRNA in a given sample was calculated from 1/2^Δ*Ct*^, in which ΔCt represents the difference between cycles required to cross the signal threshold of a target gene and those of internal control (β-actin), and then normalized with the average value of controls.

### Data Analysis

Data were expressed as means ± SEM from n numbers or pools of vessels from different animals. For statistical evaluation, a Student’s *t*-test (unpaired; two tails) was used to compare the difference between two means. When more than two means were compared, 1-way or 2-way ANOVA followed by Bonferroni’s *post hoc* test was used. *P* < *0.05* was considered statistically significant.

## Results

### Atherosclerotic Lesions, Blood Pressure and Lipids in Mice

**FIGURE 1:**
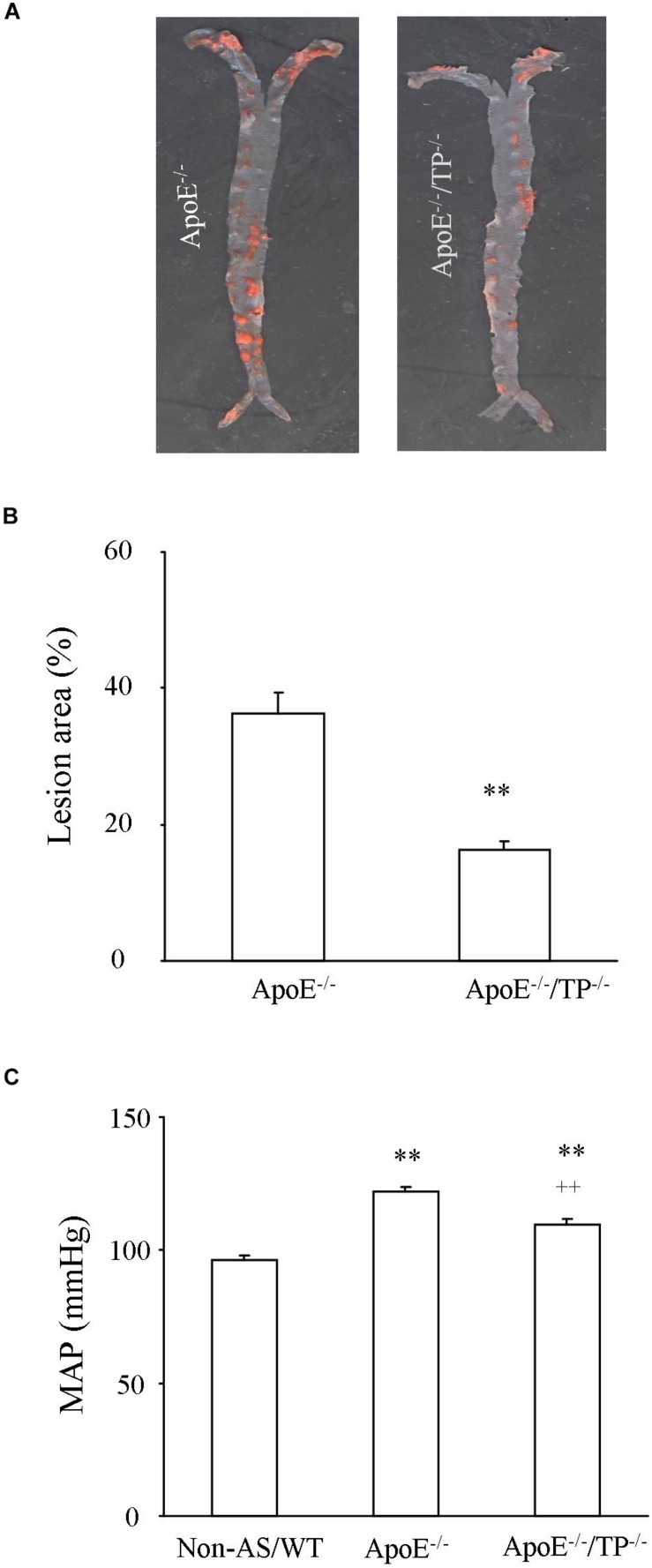
Atherosclerotic lesions and blood pressure in ApoE^–/–^ and ApoE^–/–^/TP^–/–^ mice. **(A)** Representative pictures showing the development of atherosclerotic lesions stained with Sudan IV solution (red areas) in aortas from ApoE^–/–^ and ApoE^–/–^/TP^–/–^ mice. **(B)** Summary of lesion areas relative to those of whole aortas (both thoracic and abdominal) in ApoE^–/–^ and ApoE^–/–^/TP^–/–^ mice. *N* = 8 for each group; Student’s *t*-test; *^∗∗^P* < 0.01. **(C)** MAP in ApoE^–/–^ or ApoE^–/–^/TP^–/–^ mice compared to non-atherosclerotic WT controls (Non-AS/WT). *N* = 10 for each group; 1-way ANOVA followed by Bonferroni’s *post hoc* test; *^∗∗^P* < 0.01 vs. Non-AS/WT; ^++^*P* < 0.01 vs. ApoE^–/–^.

As shown in Figures 1A,B, in ApoE^–/–^/TP^–/–^ mice atherosclerotic lesions developed in aortas, although to a lesser extent than in ApoE^–/–^ counterparts. In addition, the ApoE^–/–^/TP^–/–^ atherosclerotic mice also had an increase of blood pressure compared to non-atherosclerotic controls, and again the extent of increase was smaller than that of atherosclerotic ApoE^–/–^ mice (Figure 1C). Also, the total cholesterol level and that of LDL cholesterol were increased in atherosclerotic mice, with the extent being smaller in ApoE^–/–^/TP^–/–^ than in ApoE^–/–^ mice (Table 1).

**TABLE 1:** Blood lipids in atherosclerotic mice.

	**ApoE^–/–^**	**ApoE^–/–^/TP^–/–^**	**Non-AS/WT**
Triglyceride (mM)	1.06 ± 0.13	0.99 ± 0.08	1.14 ± 0.15
Total cholesterol (mM)	33.26 ± 1.98^∗∗^	26.00 ± 2.38^∗∗^^∧^	2.05 ± 0.15
LDL cholesterol (mM)	32.00 ± 1.87^∗∗^	23.69 ± 1.64^∗∗^^∧∧^	0.32 ± 0.05

*Values are expressed as means ± SEM; *n* = 10 for each group. Statistic was performed by 1-way ANOA followed by Bonferroni’s *post hoc* test. ^∗∗^*P* < 0.01 vs. Non-AS/WT; ^∧^*P* < 0.05 or ^∧∧^*P* < 0.01 vs. ApoE^–/–^.*

### Response to Acetylcholine in ApoE^–/–^/TP^–/–^ Atherosclerotic Rings and Effect of EP3 Blockade

**FIGURE 2:**
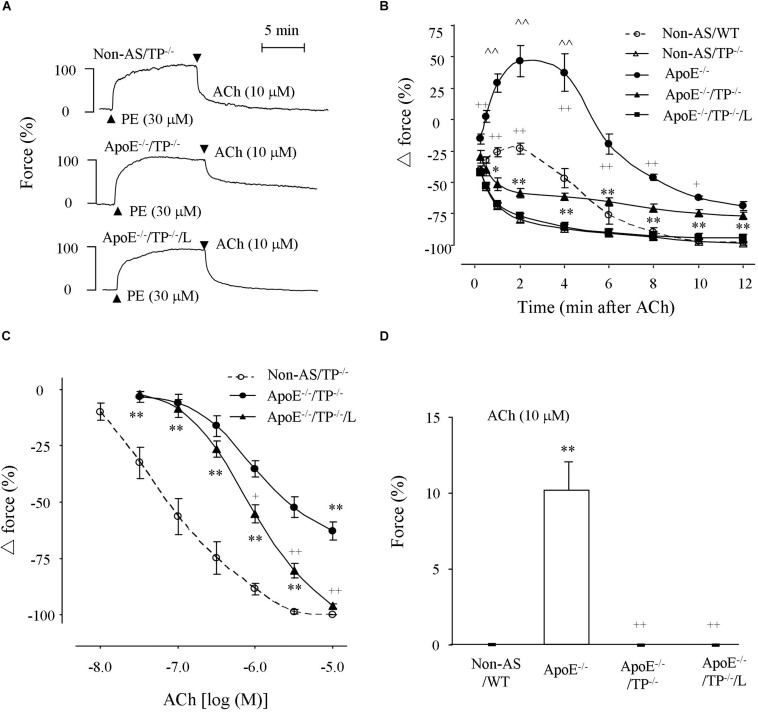
Responses evoked by acetylcholine in atherosclerotic TP^–/–^ abdominal aortic rings under NOS-uninhibited conditions. **(A)** Representative traces showing responses evoked by acetylcholine (10 μM) in phenylephrine pre-contracted non-atherosclerotic TP^–/–^ (Non-AS/TP^–/–^), atherosclerotic TP^–/–^ (ApoE^–/–^/TP^–/–^) rings and that of ApoE^–/–^/TP^–/–^ ring with the presence of L798106 (L; 1 μM; ApoE^–/–^/TP^–/–^/L). **(B)** Summary of results from **(A)** together with those of ApoE^–/–^ and Non-AS/WT. *N* = 6 for each group; 2-way ANOVA followed by Bonferroni’s *post hoc* test; *^∗^P* < 0.05 or *^∗∗^P* < 0.01 vs. Non-AS/TP^–/–^; ^+^*P* < 0.05 or ^++^*P* < 0.01 vs. ApoE^–/–^/TP^–/–^; ^∧∧^*P* < 0.01 vs. Non-AS/WT. **(C)** Concentration-dependent relaxations evoked by acetylcholine in Non-AS/TP^–/–^, ApoE^–/–^/TP^–/–^, and ApoE^–/–^/TP^–/–^/L. *N* = 5 for each group; 2-way ANOVA followed by Bonferroni’s *post hoc* test; *^∗^P* < 0.05 or *^∗∗^P* < 0.01 vs. non-AS/TP^–/–^; ^+^*P* < 0.05 or ^++^*P* < 0.01 vs ApoE^–/–^/TP^–/–^; ^∧∧^*P* < 0.01 vs. Non-AS/WT. **(D)** Responses evoked by acetylcholine (10 μM) in non-AS/WT, and ApoE^–/–^ or ApoE^–/–^/TP^–/–^ rings under baseline conditions together with that of ApoE^–/–^ rings with the presence of L (ApoE^–/–^/L). *N* = 5 for each group; 1-way ANOVA followed by Bonferroni’s *post hoc* test; *^∗∗^P* < 0.01 vs. Non-AS/WT; ^++^*P* < 0.01 vs. ApoE^–/–^.

**FIGURE 3:**
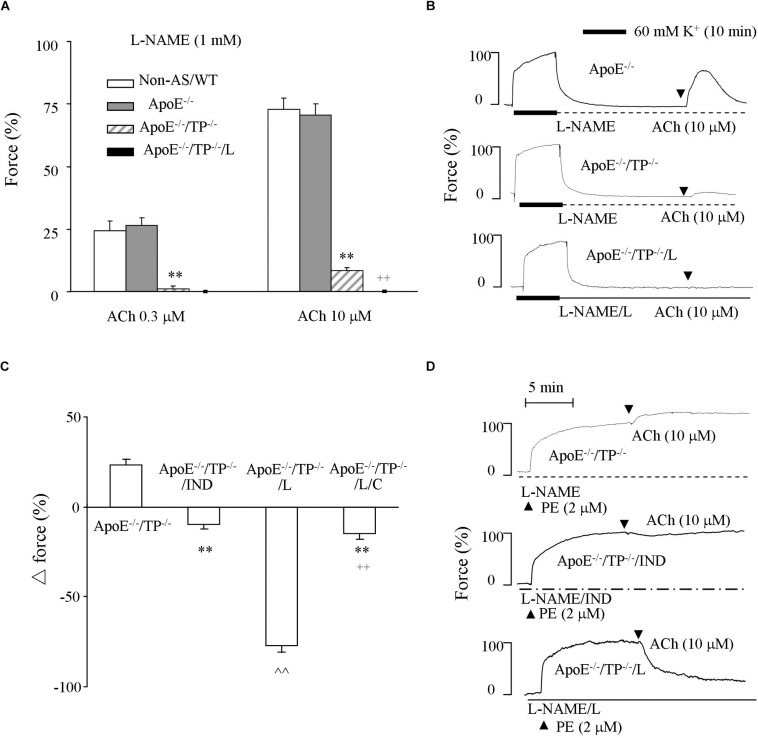
Responses evoked by acetylcholine in NOS-inhibited atherosclerotic TP^–/–^ abdominal aortic rings. **(A)** Contractions by acetylcholine (0.3 or 10 μM) in L-NAME-treated Non-AS/WT, ApoE^–/–^ or ApoE^–/–^/TP^–/–^ rings together with those of ApoE^–/–^/TP^–/–^ with the presence of L (ApoE^–/–^/TP^–/–^/L). *N* = 5 for each group; 2-way ANOVA followed by Bonferroni’s *post hoc* test; *^∗∗^P* < 0.01 vs. ApoE^–/–^; ^++^*P* < 0.01 vs. ApoE^–/–^/TP^–/–^. **(B)** Representative responses evoked by acetylcholine (10 μM) of ApoE^–/–^, ApoE^–/–^/TP^–/–^ ApoE^–/–^/TP^–/–^/L in **(A)**. **(C)** Responses evoked by acetylcholine (10 μM) in ApoE^–/–^/TP^–/–^ and those with the presence of the non-selective COX-inhibitor indomethacin (IND; 1 μM; ApoE^–/–^/TP^–/–^/IND), L (ApoE^–/–^/TP^–/–^/L), or both L and the IP antagonist CAY10441 **(C**; 1 μM; ApoE^–/–^/TP^–/–^/L/C) under L-NAME-treated, phenylephrine (2 μM) pre-contracted conditions. *N* = 5 for each group; 1-way ANOVA followed by Bonferroni’s *post hoc* test; *^∗∗^P* < 0.01 vs. ApoE^–/–^/TP^–/–^; ^∧∧^*P* < 0.01 vs. ApoE^–/–^/TP^–/–^/IND; ^++^*P* < 0.01 vs. ApoE^–/–^/TP^–/–^/L. **(D)** Representative responses of ApoE^–/–^/TP^–/–^, ApoE^–/–^/TP^–/–^/IND, and ApoE^–/–^/TP^–/–^/L in **(C)**.

**FIGURE 4:**
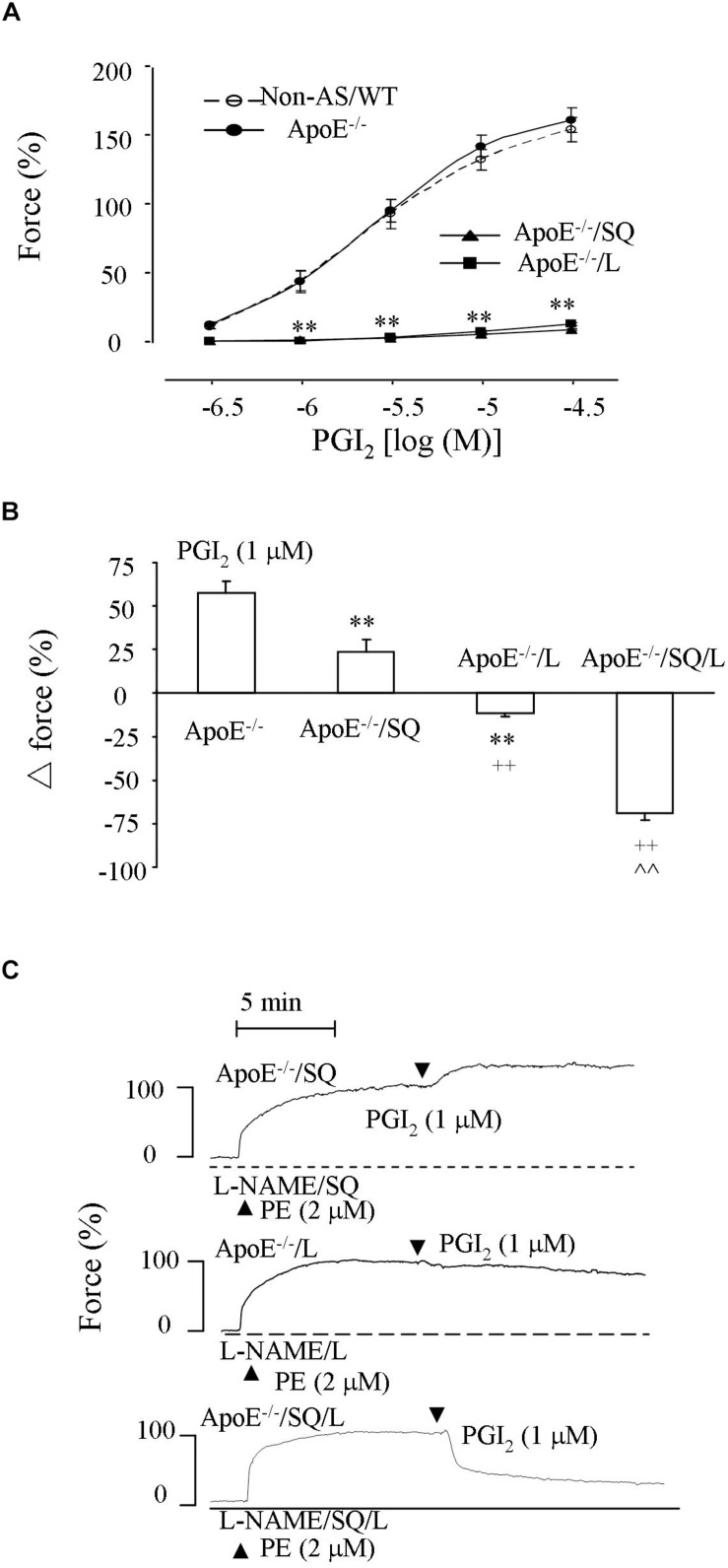
Responses evoked by PGI_2_ in NOS-inhibited atherosclerotic abdominal aortic rings. **(A)** Contractions by PGI_2_ in L-NAME-treated Non-AS/WT, ApoE^–/–^ aortic rings and those of ApoE^–/–^ obtained with the TP antagonist SQ29548 (SQ; 10 μM; ApoE^–/–^/SQ) or L (ApoE^–/–^/L). *N* = 5 for each group; 2-Way ANOVA followed by Bonferroni’s *post hoc* test; *^∗∗^P* < 0.01 vs. ApoE^–/–^. **(B)** Response evoked by PGI_2_ (1 μM) in ApoE^–/–^, ApoE^–/–^/SQ, ApoE^–/–^/L or ApoE^–/–^ rings treated with both SQ and L (ApoE^–/–^/SQ/L) under L-NAME-treated, phenylephrine (2 μM) pre-contracted conditions. *N* = 5 for each group; 1-way ANOVA followed by Bonferroni’s *post hoc* test; *^∗∗^P* < 0.01 vs. ApoE^–/–^; ^++^*P* < 0.01 vs ApoE^–/–^/SQ; ^∧∧^*P* < 0.01 vs ApoE^–/–^/L. **(C)** representative responses of ApoE^–/–^/SQ, ApoE^–/–^/L, and ApoE^–/–^/SQ/L in **(B)**.

**FIGURE 5:**
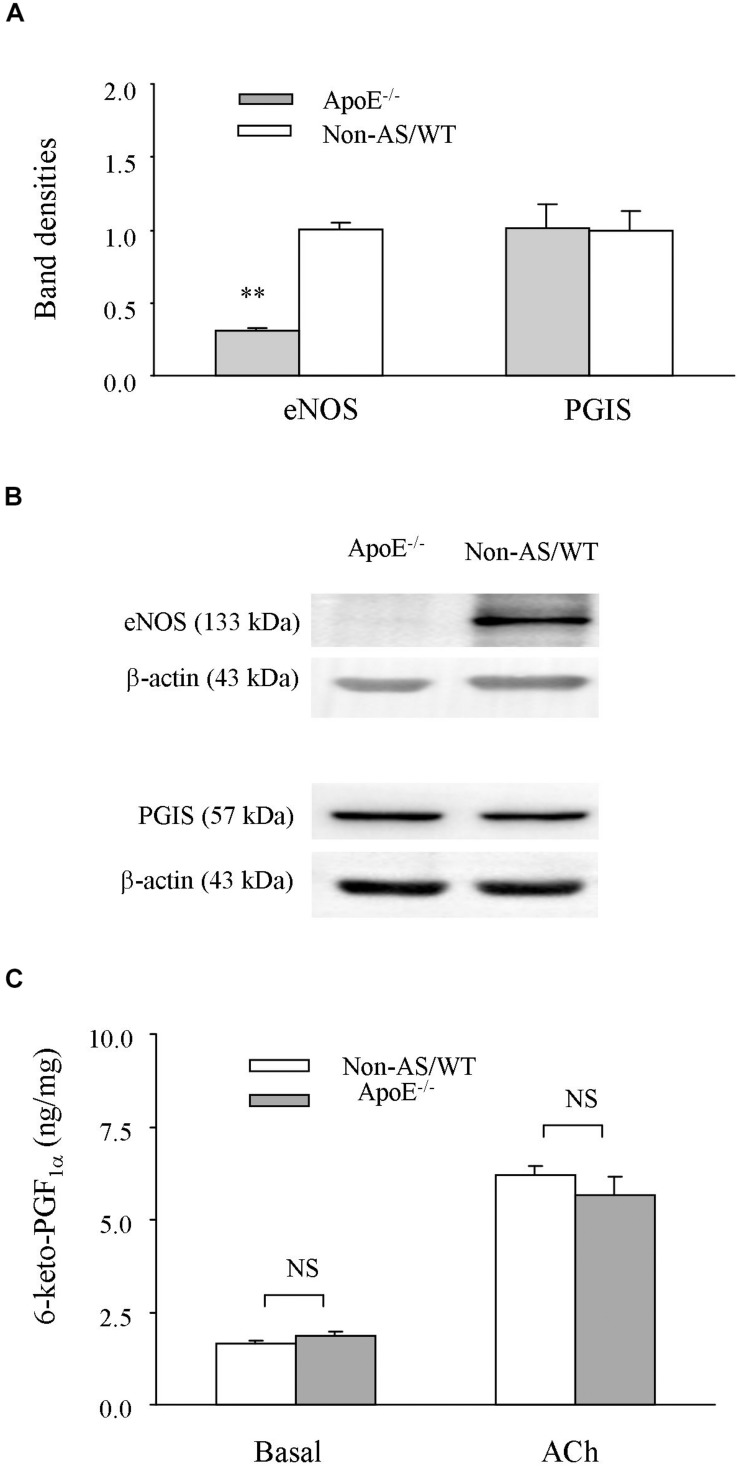
Expression of eNOS or PGIS, and production of PGI_2_. **(A,B)** A bar graph **(A)** and representative Western blots **(B)** showing expressions of eNOS, and PGIS in atherosclerotic ApoE^–/–^ abdominal aortas compared to those of non-AS/WT. In A, all values were normalized by that of β-actin and expressed relative to the average value of non-AS (*n* = 5 for each group). No significance was detected by Student’s *t*-test between ApoE^–/–^ and non-AS/WT **(C)** summary (*n* = 6 for each group) of the PGI_2_ metabolite 6-keto-PGF_1__*a*_ detected with EIA under basal (un-stimulated) or acetylcholine (10 μM) stimulated conditions in ApoE^–/–^ and Non-AS/WT. No significance was detected by 2-way ANOVA between ApoE^–/–^ and Non-AS/WT.

**FIGURE 6:**
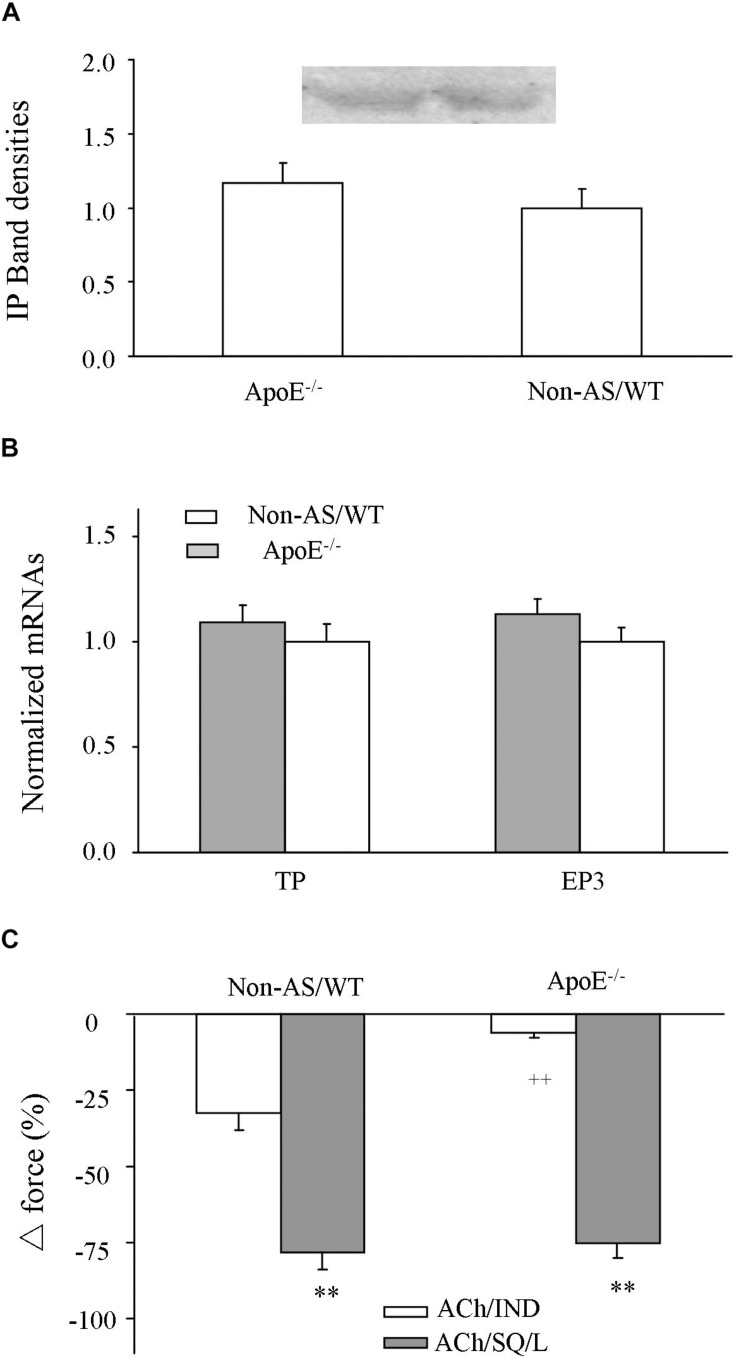
Expression of IP, EP3 or TP and NO-independent relaxation to acetylcholine after COX or TP and EP3 blockade. **(A)** Expressions of IP detected by Western blot in atherosclerotic ApoE^–/–^ abdominal aorta compared to those of Non-AS/WT. **(B)** Expressions of TP and EP3 detected by real-time PCR in atherosclerotic ApoE^–/–^ abdominal aorta compared to those of Non-AS/WT. In **(A,B)**, all values were normalized by that of β-actin and expressed relative to the average value of non-AS/WT. *N* = 5 for each group; No significance was detected by Student’s *t*-test between ApoE^–/–^ and non-AS/WT. **(C)** Relaxation evoked by acetylcholine (10 μM) in L-NAME treated, phenylephrine (2 μM) precontracted Non-AS/WT and atherosclerotic ApoE^–/–^ rings with the presence of COX inhibitor indomechanin (10 μM; ACh/IND) or of 10 μM SQ29548 and 1 μM L798106 (ACh/SQ/L). *N* = 5 for each group; 2-way ANOVA followed by Bonferroni’s *post hoc* test; *^∗∗^P* < 0.01 vs. ACh/IND; ^++^*P* < 0.01 vs. Non-AS/WT.

To study the endothelial function under atherosclerotic conditions, responses evoked by the endothelial muscarinic agonist acetylcholine were determined in vessels pre-contracted with phenylephrine (30 μM) and with NOS uninhibited. As shown in Figures 2A–C, in atherosclerotic rings of ApoE^–/–^/TP^–/–^ mice, acetylcholine evoked a relaxation, which was drastically reduced compared to that of non-atherosclerotic TP^–/–^ mice. Interestingly, the relaxation evoked by a single maximal concentration (10 μM; Figures 2A,B) or 1–10 μM of acetylcholine (Figure 2C) in atherosclerotic ApoE^–/–^/TP^–/–^ vessels was increased by the EP3 antagonist L798106 (1 μM) with the maximal response evoked by acetylcholine (10 μM) reaching a complete relaxation of phenylephrine induced contraction.

Meanwhile, an increase of force (which was larger than that of non-atherosclerotic WT vessels; with a maximal force being far above phenylephrine contraction) evoked by acetylcholine (10 μM) that blunted the concurrently activated relaxation in atherosclerotic ApoE^–/–^ rings was largely removed in ApoE^–/–^/TP^–/–^ counterparts; however, the final relaxation (12 min after 10 μM acetylcholine application) in such tissues was still comparable to that of similar ApoE^–/–^ rings and smaller than that of non-atherosclerotic WT or TP^–/–^ controls (Figure 2B and Supplementary Figure S1). It should be noted that the extents of phenylephrine pre-contractions were comparable among non-atherosclerotic WT or TP^–/–^, atherosclerotic ApoE^–/–^ or ApoE^–/–^/TP^–/–^ rings, and atherosclerotic ApoE^–/–^/TP^–/–^ rings treated with L798106 (89.1 ± 3.3%, 87.4 ± 3.1%, 91.7 ± 2.8%, 84.8 ± 3.3%, 88.9 ± 2.6%, and 85.6 + 2.9%, respectively; *n* = 6 for each group, 1-way ANOVA; *P* > 0.05).

Also, in atherosclerotic ApoE^–/–^ aortic rings but not in non-atherosclerotic controls acetylcholine (10 μM) evoked contraction under baseline conditions, similar to results we reported previously (Li et al., 2016). Again, this response was absent in atherosclerotic ApoE^–/–^/TP^–/–^ rings or in ApoE^–/–^ counterparts that had been treated with L798106 (Figure 2D).

### Responses to Acetylcholine in NOS-Inhibited Atherosclerotic Rings and Effect of EP3 Blockade

The above effect of EP3 blockade may involve a NO-independent dilator activity. To substantiate this, we performed experiments under NOS-inhibited conditions. As shown in Figures 3A,B, in L-NAME-treated ApoE^–/–^ atherosclerotic rings acetylcholine (0.3 or 10 μM) evoked contractions comparable with that of non-atherosclerotic controls. However, in ApoE^–/–^/TP^–/–^ counterparts, the contraction was significantly smaller (Figures 3A,B). Again, this remaining contraction in ApoE^–/–^/TP^–/–^ vessels was removed by the EP3 antagonist L798106 (Figures 3A,B).

The ability of acetylcholine to still evoke contraction in L-NAME-treated ApoE^–/–^/TP^–/–^ atherosclerotic rings could also be seen as an increase of force on phenylephrine (2 μM) pre-contractions (Figures 3C,D). This increase of force was reversed by the non-selective COX inhibitor indomethacin (10 μM) into a slight relaxation. Interestingly, L798106 also resulted in a relaxation to acetylcholine, which was drastically increased (>70% of phenylephrine-contraction) compared to that obtained with indomethacin (Figures 3C,D). Moreover, this increase of relaxation resulting from L798106 was drastically reduced by the IP antagonist CAY10441 (1 μM; Figure 3C).

Again, the pre-contractions to phenylephrine were not significantly different among atherosclerotic ApoE^–/–^/TP^–/–^ rings and those treated with indomethacin, L798106, or both L798106 and CAY10441 (92.2 ± 3.8%, 91.5 ± 3.9%, and 87.9 ± 3.5%, and 89.2 ± 2.9%, respectively, (*n* = 5 for each group; 1-way ANOVA; *P* > 0.05).

### Responses to PGI_2_ in ApoE^–/–^ Atherosclerotic Rings

In normal C57BL/6 abdominal aorta, PGI_2_ causes contraction reflecting the activities of TP and EP3 overcoming that of IP (Luo et al., 2016; Li et al., 2017; Liu et al., 2017). Therefore, the response evoked by PGI_2_ in NOS-inhibited atherosclerotic ApoE^–/–^ aortic rings was examined. As shown in Figure 4A, in L-NAME-treated atherosclerotic ApoE^–/–^ aortic rings PGI_2_ (0.3–30 μM) evoked a concentration-dependent contraction comparable with that in non-atherosclerotic controls yet diminished by the TP antagonist SQ29548 (10 μM) or the EP3 antagonist L798106 (1 μM).

Also, in similarly treated atherosclerotic rings pre-contracted with phenylephrine (2 μM) PGI_2_ (1 μM) evoked an increase of force, which was reduced or reversed to a slight relaxation by SQ29548 or L798106, respectively, (Figure 4B). Notably, the relaxation to PGI_2_ drastically increased when both SQ29548 and L798106 were present (Figures 4B,C). In addition, the extents of pre-contraction by phenylephrine were comparable among L-NAME-treated atherosclerotic ApoE^–/–^ aortic rings and those treated with SQ29548, L798106 or with both SQ29548 and L798106 (94.4 ± 3.5%, 92.5 ± 3.8%, and 90.9 ± 3.4%, and 88.4 ± 3.9%, respectively, (*n* = 5 for each group; 1-way ANOVA; *P* > 0.05).

### Expression of eNOS or PGIS and PGI_2_ Production in Atherosclerosis

To understand molecular bases for above results, influences of atherosclerotic development on the expression of eNOS or PGIS, and the production of PGI_2_ were examined. Western blots showed that the expression level of eNOS was lower in atherosclerotic ApoE^–/–^ abdominal aortas than in non-atherosclerotic controls, whereas those of PGIS were comparable between the two types of vessel samples (Figures 5A,B and Supplementary Figure S2). Also, we noted that either the level of PGI_2_ metabolite 6-keto-PGF_1α_ under baseline conditions (un-stimulated or in PSS) or that after acetylcholine stimulation was not significantly different between the atherosclerotic and non-atherosclerotic vessels (Figure 5C).

### Expressions of TP, EP3, and IP and NO-Independent Relaxation Evoked by Acetylcholine After TP and EP3 Blockades

Influences of atherosclerotic development on the expression level of TP, EP3 or IP and NO-independent relaxation to acetylcholine were also examined. In atherosclerotic ApoE^–/–^ abdominal aortas, the expression level of IP detected by Western blot (Figure 6A and Supplementary Figure S3) and that of TP or EP3 detected by real-time PCR (Figure 6B) were similar to those of non-atherosclerotic controls. In addition, in L-NAME-treated atherosclerotic ApoE^–/–^ aortic rings, whereas the relaxation evoked by acetylcholine after the COX inhibitor indomethacin (10 μM) was reduced, that after treatments with both the TP antagonist SQ29548 (10 μM) and the EP3 antagonist L798106 (1 μM) was similar to the response in non-atherosclerotic WT mice (Figure 6C).

## Discussion

TP blockade has been proposed to relieve endothelial dysfunction in atherosclerosis. In this study, we showed that although an increase of force evoked by the endothelial muscarinic agonist acetylcholine (larger than that of ApoE^–/–^ counterparts) that blunted the concurrently activated relaxation in ApoE^–/–^ counterparts was largely removed in atherosclerotic ApoE^–/–^/TP^–/–^ aortic rings, the relaxation evoked by the agonist in the vessels was still smaller than that of non-atherosclerotic TP^–/–^ controls. Interestingly, such a decreased relaxation in atherosclerotic ApoE^–/–^/TP^–/–^ aortic rings was increased by antagonizing EP3. These findings thus suggest a novel approach involving the removal of EP3 mediated effect along with that of TP in managing endothelial dysfunction under atherosclerotic conditions.

Analyses of lesion areas clearly showed that atherosclerosis did occur in ApoE^–/–^/TP^–/–^ mice, but to a lesser extent compared to that of ApoE^–/–^ counterparts. Also, ApoE^–/–^/TP^–/–^ mice had an improved lipid profile compared to ApoE^–/–^ counterparts. These results were consistent with a generally proposed pro-atherogenic role of TP in atherosclerotic lesion development (Kobayashi et al., 2004; Feletou et al., 2010), with part of the anti-atherogenic effect of TP^–/–^ being possibly attributed to an altered lipid profile suggested here or in a prior report with TP antagonism (Huang et al., 2018). Moreover, we noted that TP^–/–^ removed the increase of force evoked by acetylcholine (which was larger than that of non-atherosclerotic WT vessels) that blunted the concurrently activated relaxation in phenylephrine pre-contracted ApoE^–/–^ atherosclerotic vessels, concurring with a protective effect of TP antagonism on endothelial function under atherosclerotic conditions (Li et al., 2016; Romero et al., 2016; Huang et al., 2018). Indeed, this explains why the increase of blood pressure (which is frequently seen in atherosclerotic mice, and is also pro-atherogenic) was to a lesser extent in ApoE^–/–^/TP^–/–^ than in ApoE^–/–^ mice, which again can contribute to the difference of atherosclerotic lesion areas between the two mouse strains (Ross, 1999; Rader and Daugherty, 2008; Yu et al., 2009; Romero et al., 2016). It should be noted that TP^–/–^ also reduces ANG II-induced hypertension, although the effect has been suggested to originate from TxA_2_ synthesis in tissues including platelets (Kawada et al., 2004).

On the other hand, in phenylephrine pre-contracted atherosclerotic ApoE^–/–^/TP^–/–^ rings the final and maximal relaxation to acetylcholine was still comparable to the ApoE^–/–^ counterpart and smaller than that of WT or TP^–/–^ controls. This suggests a reduced endothelium-mediated dilator activity; hence, the larger increase of force evoked by acetylcholine in phenylephrine pre-contracted ApoE^–/–^ atherosclerotic rings compared to non-atherosclerotic controls could reflect a lesser extent of restraint on the EDCF activity by the concurrently activated competing endothelial dilator function, which lead to the contraction to acetylcholine seen under baseline conditions (Zhou et al., 2005; Liu et al., 2012a). In agreeing with the idea, the expression of eNOS was reduced by atherosclerotic development. Also, the smaller relaxation to acetylcholine after COX and NOS inhibitions suggests that the EDHF-mediated dilator activity is also diminished in atherosclerotic vessels. On the contrary, the expression of PGIS and production of the metabolite 6-keto-PGF_1α_ suggest that the endothelial synthesis of PGI_2_, which can act on TP and functions as an major EDCF in mouse aortas (Li et al., 2016; Luo et al., 2016), was unaltered by the atherosclerotic development. Therefore, a removal of PGI_2_’s EDCF-like action via TP without the uncovering of IP-mediated effect can explain the above effect of TP^–/–^ on the response evoked by acetylcholine under atherosclerotic conditions (Liu et al., 2012a; Luo et al., 2016; Li et al., 2017).

Interestingly, the relaxation to acetylcholine in atherosclerotic ApoE^–/–^/TP^–/–^ rings was increased by the EP3 antagonist L798106. Moreover, in such tissues, a contraction was still evoked by acetylcholine after NOS inhibition, but it was reversed by L798106 into a relaxation sensitive to IP antagonism. Thus, TP^–/–^ indeed only resulted in a partial removal of EDCF activity and the above effects of L798106 in atherosclerotic ApoE^–/–^/TP^–/–^ rings reflected an abolition of the remaining EP3-mediated EDCF activity that outweighed the effect of IP, leading to an uncovering of the dilator action of natively produced PGI_2_ that added to the reduced endothelial dilator function in atherosclerotic conditions. Consistent with the idea, PGI_2_ still evoked contraction in atherosclerotic rings after TP blockade, but relaxation when both TP and EP3 had been antagonized. It should be noted that the EP3 antagonist L798106 is also a partial antagonist of TP (Liu et al., 2017). This could be the reason why L798106 inhibited PGI_2_-evoked contraction to an extent similar to that of the TP antagonist SQ29548 or resulted in a slight relaxation to PGI_2_ under phenylephrine pre-contracted conditions, although the vasoconstrictor activity of EP3 could be smaller than that of TP, as reflected by the effect of respective gene deletion (Li et al., 2017; Liu et al., 2017).

To date, TP blockade has been considered an effective way to remove EDCF activity and relieve endothelial dysfunction in diseases, including atherosclerosis (Gluais et al., 2005; Vanhoutte, 2009, 2011; Li et al., 2016; Romero et al., 2016; Huang et al., 2018). Interestingly, one of our recent reports suggests that PGI_2_, a major EDCF, may act on both TP and EP3 to overcome the dilator effect of concurrently activated IP and result in a contractile response to the agonist (Li et al., 2017). Results from the present study further reveal that in atherosclerotic TP^–/–^ vessels, EP3 blockade not only abolishes the remaining EDCF activity, but also leads to an uncovering of the IP-mediated dilator action resulting from natively produced PGI_2_ that can add to the reduced endothelial dilator function under the disease condition. It should be noted that mRNA detection and PGI_2_ responses suggest that expressions of EP3 and TP were unaltered by atherosclerotic development. Other PGs (e.g., PGF_2α_ and PGE_2_) and isoprostanes, which could be released along with PGI_2_ although in smaller amounts, also evoke contraction via TP and EP3 (Janssen and Tazzeo, 2002; Gluais et al., 2005; Wong et al., 2009; Li et al., 2016, 2017; Liu et al., 2019). In addition, EP3 implicates in inflammation and atherothrombosis (Gross et al., 2007; Morimoto et al., 2014; Mawhin et al., 2015). These results together underscore the importance of targeting EP3 along with that of TP in atherosclerotic conditions. Notably, PGI_2_ may evoke contraction in coronary circulation (Dusting et al., 1977). A contraction to acetylcholine also occurs in atherosclerotic human coronary arteries (Ludmer et al., 1986; Yasue et al., 1986; Okumura et al., 1988; Sueda et al., 2015). Thus, further studies are needed to determine the pathological relationship of PGI_2_ with TP and/or EP3 in ischemic heart disease, since the contraction evoked by acetylcholine originally considered to originate from smooth muscle could in fact be an endothelium-mediated response under disease conditions (Tesfamariam et al., 1989; Srisawat et al., 2003).

In contrast to findings of the present study, productions of PGI_2_ and TxA_2_ have been suggested to increase in atherosclerotic vessels (FitzGerald et al., 1984; Pratico et al., 2001; McClelland et al., 2009). However, in the present study, PGI_2_ was expressed against vessel weight, which is larger in atherosclerotic than in non-atherosclerotic conditions. We and others have shown that acetylcholine does not stimulate TxA_2_ production in conditions, including atherosclerosis (Wong et al., 2009; Vanhoutte, 2011; Liu B. et al., 2015; Li et al., 2016). Thus, TxA_2_ could not be an EDCF under the disease conditions, although minor involvements of other PGs and isoprostanes could not be excluded as discussed above. There are also studies suggesting that EDHF effect increases in atherosclerosis (Ozkor and Quyyumi, 2011; Csanyi et al., 2012). This could result from difference in stages of the disease. For example, we have previously shown that the relaxation to acetylcholine after COX and NOS inhibitions in vessel areas without atherosclerotic lesions is similar to that of non-atherosclerotic controls (Li et al., 2016). We noted that in NOS-inhibited atherosclerotic rings where EDHF appeared to be minimal, the relaxation to acetylcholine after TP and EP3 blockades under NOS inhibited conditions was comparable to that of non-atherosclerotic controls. An explanation could be that the relaxation by EDHF or IP shares a common pathway (Hellsten et al., 2012; Liu B. et al., 2015), and hence the resulted response is determined by the larger one mediated by IP, whose function or expression was again unaltered by atherosclerotic development as seen from PGI_2_-evoked responses and protein detection of the receptor. It should be noted that although the unaltered dilator action of IP in atherosclerotic vessels of the present study might include a facilitating effect of ApoE^–/–^ suggested previously (Cheng et al., 2018), a full uncovering of IP-mediated effect would still be beneficial to the reduced endothelial dilator function, even it might be reduced in atherosclerotic conditions.

In summary, in this study our results demonstrate that EP3 blockade adds to the effect of TP^–/–^ in uncovering the dilator action of natively produced PGI_2_ that can alleviate endothelial dysfunction in atherosclerotic conditions.

## Data Availability Statement

All datasets generated for this study are included in the manuscript/Supplementary Files.

## Ethics Statement

Mice were housed in the animal facility of Shantou University Medical College under standard conditions with free access to water and food. All procedures performed on mice were in conformance with the Guide for the Care and Use of Laboratory Animals published by the US National Institutes of Health (NIH Publication No. 85-23, revised 1996), and approved by the Institutional Animal Research and Use Committee of Shantou University.

## Author Contributions

CH, BL, YX, XW, TG, YzZ, JL, JaG, GY, and JnG performed the research. CH, BL, and YbZ analyzed the data. BL and YbZ designed the research and wrote the manuscript. All authors contributed to the manuscript revision, and read and approved the submission.

## Conflict of Interest

The authors declare that the research was conducted in the absence of any commercial or financial relationships that could be construed as a potential conflict of interest.
