# Supplementary figures and images for: EP3 Blockade Adds to the Effect of TP Deficiency in Alleviating Endothelial Dysfunction in Atherosclerotic Mouse Aortas
Source: Front Physiol. 2019 Sep 26;10:1247. doi: 10.3389/fphys.2019.01247 (PMC6775864; doi:10.3389/fphys.2019.01247)

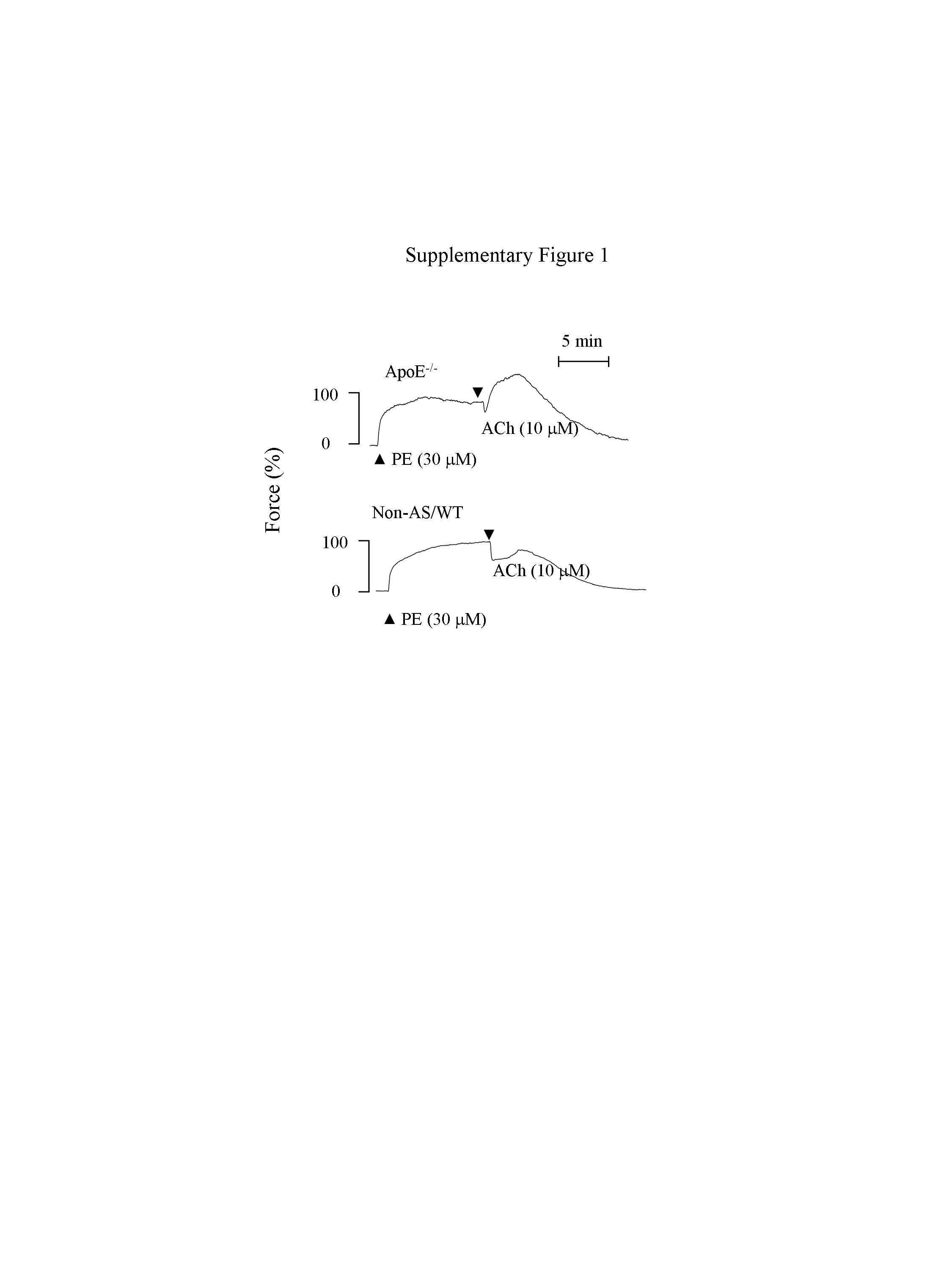

Supplement: Supplementary file 1 [file Image_1.JPEG]

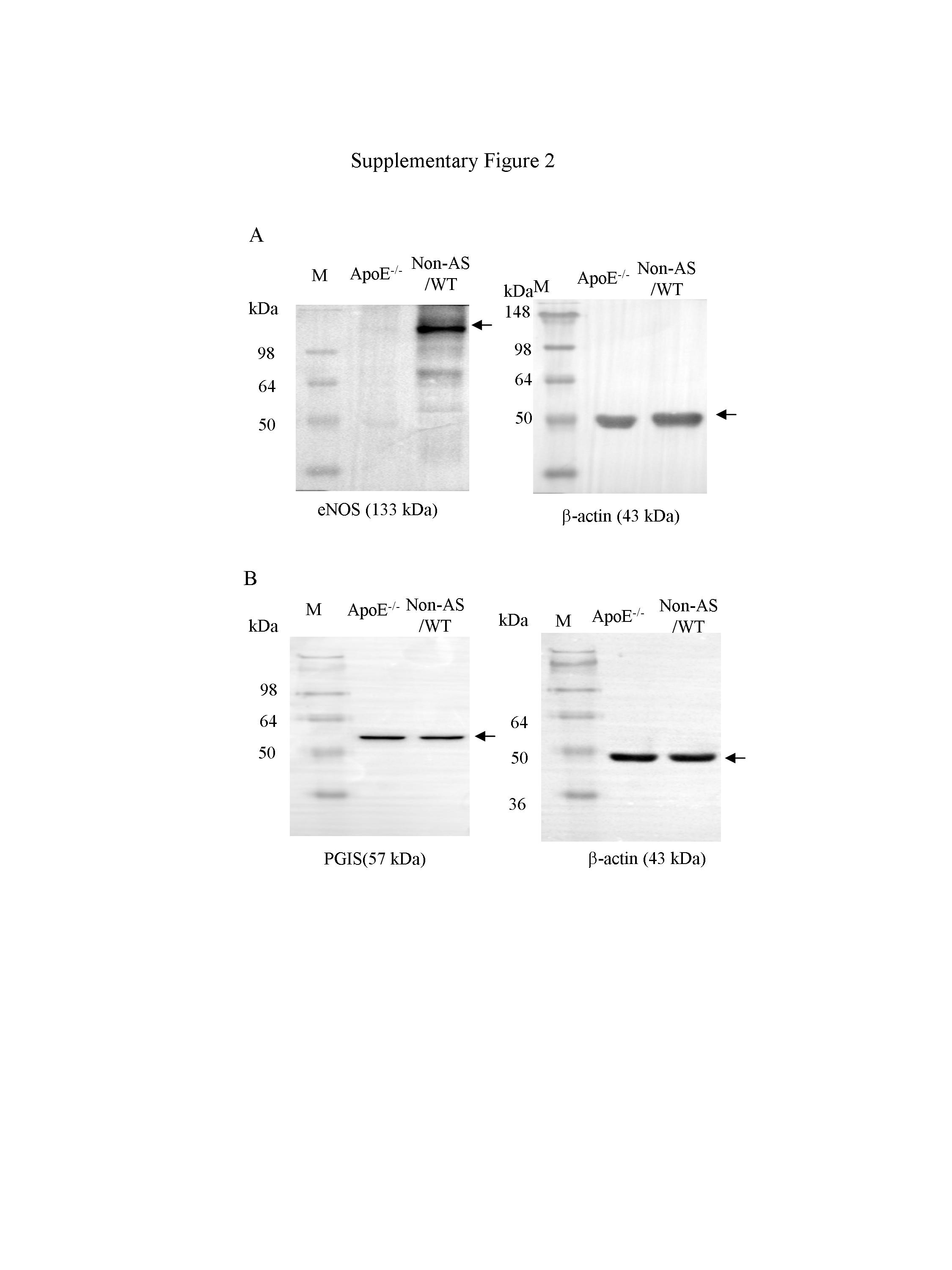

Supplement: Supplementary file 2 [file Image_2.JPEG]

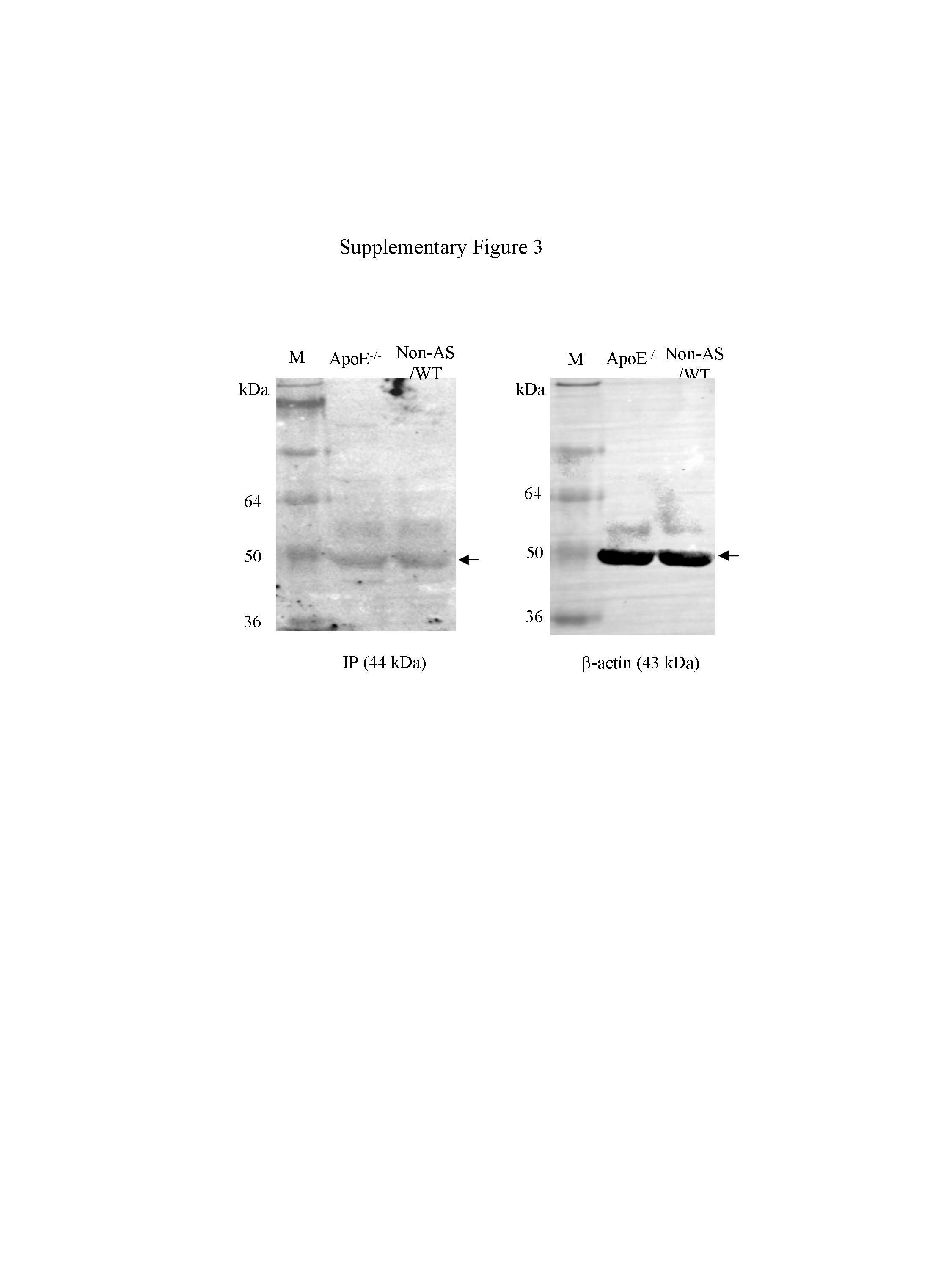

Supplement: Supplementary file 3 [file Image_3.JPEG]
