# Supplementary material for: EP3 Blockade Adds to the Effect of TP Deficiency in Alleviating Endothelial Dysfunction in Atherosclerotic Mouse Aortas
Source: Front Physiol. 2019 Sep 26;10:1247. doi: 10.3389/fphys.2019.01247 (PMC6775864; doi:10.3389/fphys.2019.01247)
Supplement: Supplementary file 4 [file Data_Sheet_1.doc]

**Figure Legends for Supplementary Data**

**Supplementary Figure 1.** Representative traces showing the response evoked by ACh (10 μM) in PE (30 μM) pre-contracted atherosclerotic ApoE-/- (ApoE-/-) rings and that of non-atherosclerotic WT controls (Non-AS/WT) in Figure 2 of the MS.

**Supplementary Figure 2.** Full length gels of representative Western blots of Fig 5, on which eNOS (A) or PGIS (B) were blotted after β-actin bands (right) were striped off using a 62.5 mM Tris·HCl buffer (pH 6.8) containing 2% SDS, and 0.8% β-mercaptoethanol (1). The expected bands were indicated with arrows. M: size makers

**Supplementary Figure 3.** A full length gel of representative Western blots of Fig 6, on which β-actin (right) was further probed after bands of IP were striped off using a 62.5 mM Tris·HCl buffer (pH 6.8) containing 2% SDS, and 0.8% β-mercaptoethanol (1). The expected bands were indicated with arrows. M: size markers

**Supplementary Reference**

1. Kaufmann SH, Ewing CM, Shaper JH. The erasable Western blot. Anal Biochem 161: 89 –95, 1987
